# A Novel Bird Sound Recognition Method Based on Multifeature Fusion and a Transformer Encoder

**DOI:** 10.3390/s23198099

**Published:** 2023-09-27

**Authors:** Shaokai Zhang, Yuan Gao, Jianmin Cai, Hangxiao Yang, Qijun Zhao, Fan Pan

**Affiliations:** 1 College of Electronics and Information Engineering, Sichuan University, Chengdu 610041, China; 2020141420292@stu.scu.edu.cn (S.Z.); 2020141450074@stu.scu.edu.cn (Y.G.); 2021222050149@stu.scu.edu.cn (J.C.); 2 College of Computer Science, Sichuan University, Chengdu 610041, China; 2020141420288@stu.scu.edu.cn (H.Y.); qjzhao@scu.edu.cn (Q.Z.)

**Keywords:** bird sound recognition, feature fusion, multiple acoustic features, biodiversity

## Abstract

Birds play a vital role in the study of ecosystems and biodiversity. Accurate bird identification helps monitor biodiversity, understand the functions of ecosystems, and develop effective conservation strategies. However, previous bird sound recognition methods often relied on single features and overlooked the spatial information associated with these features, leading to low accuracy. Recognizing this gap, the present study proposed a bird sound recognition method that employs multiple convolutional neural-based networks and a transformer encoder to provide a reliable solution for identifying and classifying birds based on their unique sounds. We manually extracted various acoustic features as model inputs, and feature fusion was applied to obtain the final set of feature vectors. Feature fusion combines the deep features extracted by various networks, resulting in a more comprehensive feature set, thereby improving recognition accuracy. The multiple integrated acoustic features, such as mel frequency cepstral coefficients (MFCC), chroma features (Chroma) and Tonnetz features, were encoded by a transformer encoder. The transformer encoder effectively extracted the positional relationships between bird sound features, resulting in enhanced recognition accuracy. The experimental results demonstrated the exceptional performance of our method with an accuracy of 97.99%, a recall of 96.14%, an F1 score of 96.88% and a precision of 97.97% on the Birdsdata dataset. Furthermore, our method achieved an accuracy of 93.18%, a recall of 92.43%, an F1 score of 93.14% and a precision of 93.25% on the Cornell Bird Challenge 2020 (CBC) dataset.

## 1. Introduction

Bird species protection plays a vital role in biodiversity conservation [1,2]. The abundance of bird populations directly reflects local biodiversity levels, emphasizing the importance of bird recognition in ecological research and biodiversity conservation. Bird sound-based recognition methods offer significant advantages over other bird recognition methods that are commonly used in ornithological studies, such as image-based methods and the marked-recapture method [3]. They have wider recognition ranges, are immune to forest obstructions, and experience reduced interference from human activities, making them the preferred solutions for bird recognition [4].

In recent years, bird populations have experienced a noticeable decline due to human and environmental factors. Morrison et al. [5] conducted a study that presented compelling evidence of a substantial decrease in the diversity and intensity of bird soundscapes across over 200,000 sites in the Northern Hemisphere over the past 25 years. This decline can be attributed to significant decreases in bird species and individual abundance, which have far-reaching consequences on ecosystem health and biodiversity. Therefore, swiftly and accurately recognizing birds through sound analysis is critical for bird population monitoring and ecological conservation efforts.

Traditional bird sound-based methods for bird species recognition, which rely on manual differentiation, suffer from subjectivity and lack reliable verifications [6]. With advancements in machine learning techniques, the field of bird sound recognition has shifted from manual differentiation to machine learning implementation. Generally, there are two types of implementation approaches for bird sound recognition based on machine learning [7]. The first type of approach is template matching [8], such as the dynamic time warping (DTW) algorithm [9]. The second approach involves feature-based recognition methods, including Gaussian mixture models [10], hidden Markov models [11], support vector machines [12] and random forests [13,14]. The application of these machine learning-based methods mitigates the effect of human perceptual bias on bird sound recognition to some extent [6]. However, when confronted with highly variable and complex bird sounds, these methods often struggle to achieve high recognition rates. These challenges call for the development of more robust and accurate techniques to attain improved bird sound recognition performance.

Recently, deep learning methods have gained significant popularity in the field of bird sound recognition due to their ability to automatically extract features from inputs [15] and their greater robustness against environmental noise [16]. As most bird sounds are recorded in the field, where ambient noise is significant [12,17], quantile-based noise estimation techniques such as spectral subtraction [18] and silence detection [19] are commonly applied during data preprocessing to mitigate the impacts of noise and silent segments on bird sound recognition. The continuous development of deep learning architectures, including convolutional neural networks (CNNs) [20,21] and recurrent neural networks (RNNs) [22], has solidified their position as popular choices for bird sound recognition. The study conducted by [23] demonstrated the superiority of deep learning methods over traditional machine learning methods in the field of bird sound recognition. For instance, Sankupellay and Konovalov [24] employed ResNet50 for automatic bird sound recognition using spectrograms obtained from the sounds of 46 distinct bird species as inputs [7], achieving a maximum accuracy of 72%.

In recent years, transformers have also garnered significant attention in the field of bird sound recognition. Puget [25] introduced a transformer-based bird sound recognition model, the STFT-Transformer, which utilizes log-mel spectrograms as transformer inputs for bird sound recognition. The results demonstrated that the STFT-Transformer outperformed traditional CNNs in terms of accuracy and speed. Tang et al. [26] employed a ViT model to encode visual features based on a multihead attention mechanism. They emphasized that the multihead attention mechanism allows their model attending to information derived from various locations and features, facilitating the extraction of bird sound features across multiple dimensions. Gunawan et al. [27] employed an efficient attention mechanism called the CBAM to encode the spatial information within bird sound features. This mechanism incorporates both channel attention and spatial attention modules. The utilization of the spatial attention module can complement the spatial perception capability of the channel attention module, thus efficiently capturing the spatial information contained in the bird sound features. This is in contrast to transformer models that rely on a multihead attention mechanisms, which can more easily capture global information as well as spatial positional relationships in bird sound features because the attention is computed across all inputs [28]. Many studies have demonstrated that utilizing a combination of acoustic features for bird sound recognition outperforms the use of only a single feature set [29]. Xiao et al. [30] employed the MFCC-CST feature set, which includes MFCC, Chroma, spectral contrast, and Tonnetz features. Utilizing these features, they trained an AMResNet network with an attention mechanism and achieved an impressive accuracy of 90.1% on the Birdsdata dataset. The experimental results indicated that this combination of features was efficient. Similarly, Hidayat et al. [31] achieved a remarkable accuracy of 96.08% on a dataset comprising seven bird species by combining log-mel spectrograms and MFCC features. According to [32,33], visual representations of the acoustic features derived from bird sounds have been demonstrated to be effective in bird sound recognition tasks, as spectrograms can adeptly capture the spatial information that is present in sounds. Based on their study, we use MFCC, chroma and Tonnetz features from the MFCC-CST feature set [13] and log-mel spectrograms [31] as model inputs.

The previously mentioned AMResNet [30], which is derived from residual networks and incorporates attention layers, achieves high recognition accuracy on the Birdsdata dataset. However, the attention layer used by AMResNet needs to be added manually, which complicates the model construction process. Furthermore, Adavanne et al. [34] argued that CNNs overlook the distinctive characteristics of bird sounds as time-series signals, thus failing to capture comprehensive features. Furthermore, bird sounds often display temporal discontinuities, with the intervals between bird sounds lasting several seconds [35]. However, the translational invariance property of CNNs presents challenges when capturing the positional relationships among these disconnected features [36]. To address this challenge, we combine CNN-based networks with a transformer encoder to leverage the CNN-based networks’ powerful spectrogram texture feature extraction capabilities [7] and compensate for their shortcomings in terms of capturing long-term contextual spatial information from feature sequences [36].

This study makes a significant contribution by introducing a novel bird sound recognition method. Our method combines two CNN-based networks and an improved transformer encoder, incorporating various acoustic bird sound features. Feature fusion is employed to obtain more comprehensive feature information [23]. The proposed method effectively leverages the strengths of CNN-based networks in terms of extracting log-mel spectrogram features [7] while utilizing the transformer encoder’s capabilities to capture the long-term contextual information contained in feature sequences [28]. Log-mel spectrograms are fed into pretrained EfficientNetB3 [37] and ResNet50 [38] models to obtain a fused feature vector. Additionally, an improved transformer encoder is utilized to encode the MFCC, Chroma, and Tonnetz feature sets, generating an additional set of feature vectors. Subsequently, these two feature vectors are fused and utilized for classification using a light gradient boosting machine (LightGBM) [39]. This method allowed us the extraction of distinctive features from different networks and their fusion, leveraging the CNN-based networks’ capability to extract image features and the transformer’s proficiency in processing long sequences. As a result, the model’s feature extraction capacity was increased, leading to improved classification accuracy. The method proposed in this paper achieves 97.99% and 93.18% accuracy on two publicly available datasets, the Birdsdata dataset and the CBC dataset, respectively. The experimental results demonstrate the great potential of this method for applications in biodiversity monitoring, animal acoustics research, and ecological environmental protection. The rest of this paper is organized as follows. Section 2 provides a detailed description of the model and methodology employed in this study. Section 3 presents the experimental results and evaluates the performance of the proposed model. Finally, in Section 4, we conclude the paper, discuss the study’s practical implications, and suggest potential directions for future research.

## 2. Materials and Methods

**Figure 1:**
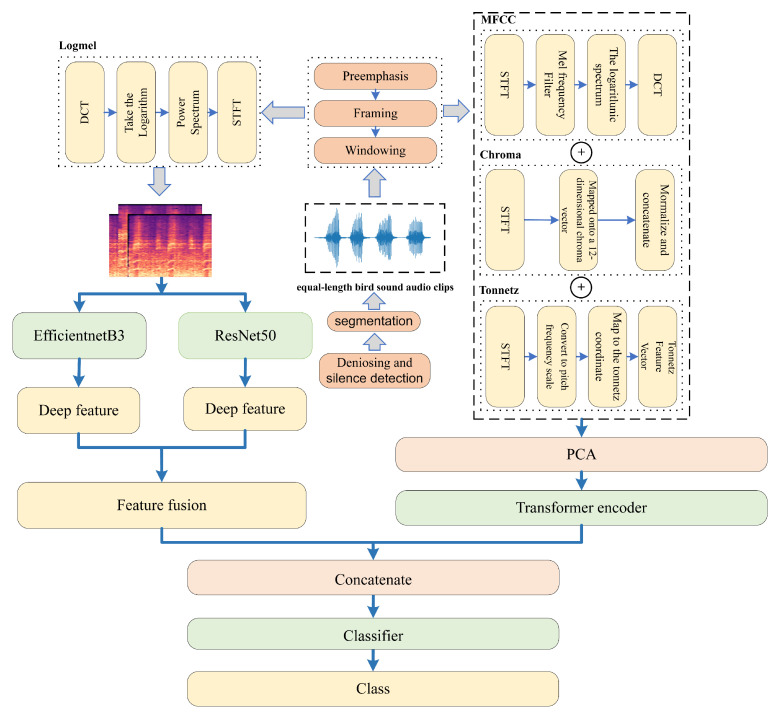
The process of the proposed method. The bird sounds were preprocessed, and the manually extracted log-mel spectrograms were then fed into two pretrained CNN-based networks to acquire a set of deep features. Three more manually extracted features (MFCC, Chroma, and Tonnetz features) were combined, forming a feature set that was subsequently encoded by an improved transformer encoder. Finally, both resulting deep feature vectors were fused and passed to a classifier for classification.

This section presents a detailed description of the proposed method employed in this paper and the two public datasets used. The preprocessing steps involve performing noise reduction and silence detection on bird sound fragments. Next, we introduce acoustic bird sound features as model inputs. Additionally, we offer comprehensive explanations of the deep feature extraction process and the specific implementation of the feature fusion approach. A LightGBM is used as the classifier. The specific process is illustrated in Figure 1.

### 2.1. Dataset

High-quality bird sound recognition studies require reliable, extensive, and accurate publicly available datasets. In this study, we utilized two such datasets that are publicly available: the Beijing Hundred Birds dataset (Birdsdata) and the Cornell Bird Challenge 2020 (CBC) dataset.

#### 2.1.1. Birdsdata

The Birdsdata dataset, curated and collected through a collaboration between the Beijing Zhiyuan Institute of Artificial Intelligence and Hundred Birds Data, is highly regarded for its credibility and precision [26,30]. The dataset comprises recordings of 20 prevalent bird species. All bird sound clips underwent rigorous noise reduction and were trimmed to uniform lengths. Consequently, no further data preprocessing was applied. With 14,311 bird sound clips each lasting 2 s, approximately 90% of the species possessed over 300 samples, while approximately 5% of the species included less than 50 samples, ensuring comprehensive representations; https://data.baai.ac.cn/details/Birdsdata, accessed on 28 June 2023.

#### 2.1.2. Cornell Bird Challenge 2020 (CBC) Dataset

The Cornell Bird Challenge 2020, organized by the Cornell Lab of Ornithology at Cornell University, is a bird sound recognition challenge aimed at advancing the technology in this field and promoting collaboration among researchers and developers. The CBC dataset, provided by the Cornell Lab of Ornithology, consists of a diverse collection of bird sound recordings from various bird species worldwide. In total, the dataset contains vocalizations from 264 bird species. The original audio files in the CBC dataset were in the MP3 format but were converted to the WAV format for more efficient processing and analysis in this study. To ensure consistency across the data, normalization techniques were applied by considering the varied durations of the raw audio files [40]. Through the application of noise reduction, silence detection, and segment cutting techniques, we obtained 114,287 bird song segments, each lasting 5 s. Approximately 72% of the bird species in the dataset had over 300 samples, while only approximately 3.4% had fewer than 50 samples. https://www.kaggle.com/c/birdsong-recognition/data, accessed on 16 June 2023.

#### 2.1.3. Partitioning of the Dataset

The extensive use of these two datasets [26,30,41], along with the high-quality data samples, ensured that our study had a reliable and accurate database. The length of the bird sound segments in the Birdsdata dataset was 2 s. However, in the CBC dataset, where a larger number of bird species were present, the bird sound segments processed through denoising and silence detections were uniformly trimmed to 5 s to ensure that each bird sound segment encompassed sufficient information. We standardized the dimensionality of the manually extracted bird sound features to counter the impact of the varying bird sound segment lengths between the Birdsdata and CBC datasets on the model’s performance. To train, validate, and test our models effectively, we divided each dataset into three subsets: a training set, a validation set, and a testing set. Table 1 provides specific details about teh distribution of the data, with 60% of the samples assigned to the training set and 20% allocated to each of the validation set and testing set. During the partitioning process, we employed a stratified sampling approach to maintain a proportional distribution of bird species categories across the training, validation, and testing sets. This approach ensured that the same category distribution observed in the entire dataset was preserved.

### 2.2. Data Preprocessing

To ensure accurate bird sound recognition, preprocessing the audio recordings collected in real-world environments is essential [14,17,19]. This is due to the nonsmooth characteristics of bird sounds and the presence of environmental noise [7].

#### 2.2.1. Denoising

**Figure 2:**
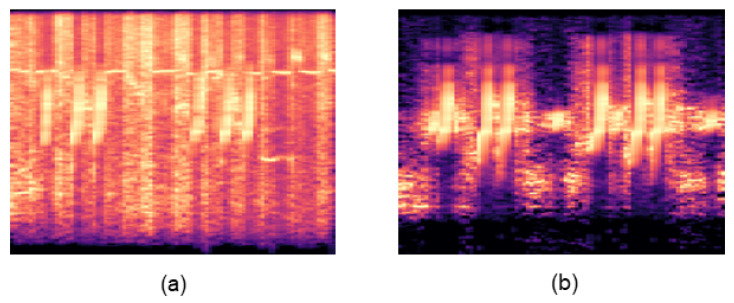
(**a**) The log-mel spectrogram of the sound produced by a common quail; (**b**) the log-mel spectrogram of the sound produced by the common quail after applying denoising. The effectiveness of the quantile-based noise estimation method for spectral subtraction used in this study is evident in the elimination of the noise present in the bird vocalizations.

To enhance the accuracy of bird sound recognition in real-world environments, the application of denoising techniques to obtain cleaner bird sound segments is crucial [12]. In this study, we utilized a quantile-based noise estimation method for spectral subtraction to reduce the noise in the bird sound segments [18]. The method automatically estimated the noise within each segment by leveraging the inherent characteristics of bird sounds, allowing for efficient noise removal while minimizing interference with the bird sound signals. The effectiveness of this approach is demonstrated in Figure 2. We started the denoising process by applying a high-pass filter to eliminate low-frequency noise from the bird sounds. Next, we calculated the power spectrum of the bird sound signal and employed a quantile noise estimation method [18] to determine the noise threshold. To obtain a reliable noise estimate, an appropriate quantile was selected, and median filtering was applied to the estimated noise values of consecutive frames. This smoothing process enhanced the stability of the noise estimation process. The noise threshold was calculated as

(1) T(f)=α·median{P(f,n)}.

The chosen quantile for noise estimation is represented by α, and P(f,n) indicates the frequency of *f* at the *n*th power spectrum value.

A mask threshold was calculated to eliminate the silent frames in the bird sounds that had values smaller than the noise threshold. Subsequently, the corrected power spectrum was recalculated using the mask threshold. To reconstruct the noise-reduced bird sound signals, we conducted an inverse short-time Fourier transform with the phase information of the bird sounds. This process effectively removed noise while preserving the valuable bird sounds due to the adaptive adjustment of the mask threshold based on the statistical characteristics of the bird sound signals. Due to its adaptability and efficiency, this method for reducing bird sound noise is suitable for real-time application in field bird sound recordings and can be employed in embedded environments [42].

#### 2.2.2. Silence Detection and Segmentation

To improve the accuracy of bird sound recognition by mitigating the impact of silent segments inside the bird sound fragments, the average envelope threshold method was utilized for silence detection in the bird sound fragments. This method analyzed the average envelope of the frames within a sliding window to determine whether a fragment corresponded to a silent portion. By eliminating the silent frames, only the valid bird sounds remained, enabling a more focused analysis of the bird sounds. The method can be described using the following equation:

(2) $$\begin{array}{cc} \; & y_{\mathrm{mean}\;}\left[n\right]=\frac{1}{L}\sum_{k=-\frac{L-1}{2}}^{\frac{L-1}{2}}\left|y\left[n+k\right]\right|,\\ \; & mask\left[n\right]=\left\{\begin{array}{cc} \;\mathrm{True}\; & \;\mathrm{if}\;y_{\mathrm{mean}\;}\left[n\right]>\;threshold\;\\ \;\mathrm{False}\; & \;\mathrm{otherwise}\; \end{array}\right.. \end{array}$$

In this equation, y[n] represents the first *n* frames of the bird sound signal, *L* denotes the length of the sliding window, $y_{\mathrm{mean}\;}\left[n\right]$ denotes the average envelope within the window, mask[n] determines whether the first *n* frame is silent or not, and threshold is the predefined threshold value. Subsequently, the processed bird sound files were divided into equal-length segments.

### 2.3. Construction of the Input Features

Previous studies [30,31] have demonstrated the effectiveness of incorporating aggregated features into bird sound recognition, leading to improved classification performance. Building upon these findings, our method adopted manually extracted log-mel spectrograms and MFCC, Chroma, and Tonnetz features. By utilizing these diverse acoustic features, our goal was to capture a comprehensive representation of the bird sounds, ultimately improving the performance of our model.

#### 2.3.1. Log-Mel Spectrograms

**Figure 3:**
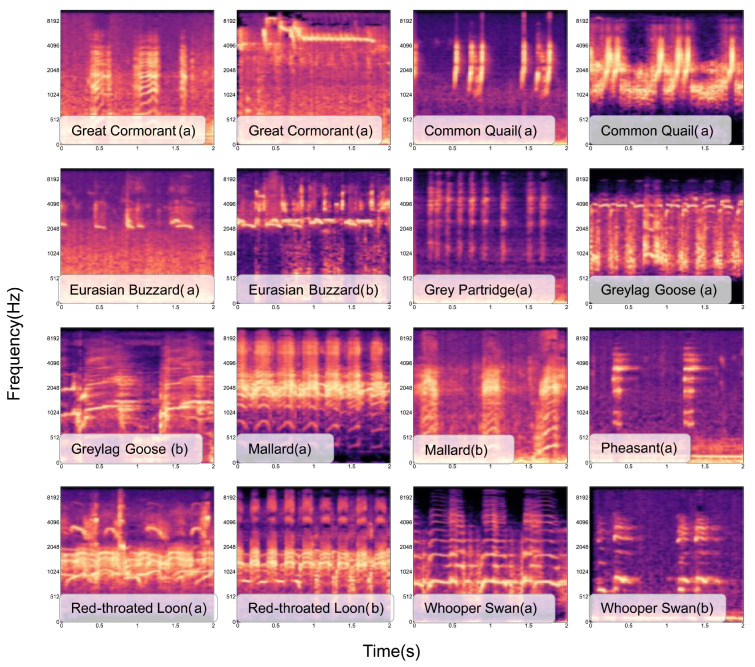
The log-mel spectrograms associated with specific labels. (a) and (b) depict different vocalizations of the same bird species. The figure shows that each bird species displays distinct texture features in its corresponding log-mel spectrogram. Thus, these log-mel spectrograms can serve as a foundation for bird sound recognition.

A log-mel spectrogram of bird sounds provides a visual representation of how the frequency components of bird sounds change over time using a logarithmic scale [7]. First, we applied the short-time Fourier transform (STFT) [43] to the bird sound fragments to obtain a spectrogram. Subsequently, we used a filter bank with 128 frequency bands to map the spectrogram to the mel scale because it aligns better with the human auditory system’s perception and is more suitable for bird sound feature extraction [44]. Then, we performed a logarithmic transformation to obtain the log-mel spectrogram. The log-mel spectrogram holds significant biological relevance and interpretability and possesses extensive applications in various fields, including sound recognition and audio classification [45]. The log-mel spectrograms of selected bird sound clips are shown in Figure 3.

#### 2.3.2. MFCC, Chroma, and Tonnetz Features

Mel frequency cepstral coefficients (MFCCs) [46] have found wide applications in audio signal processing and are commonly utilized in bird sound recognition [47]. MFCCs simulate the functioning of the human auditory system by transforming a bird sound signal from the time domain to the frequency domain and employing mel filters for filtering. The resulting filtered signal is then subjected to a discrete cosine transform (DCT) [48], generating a set of MFCCs that serve as a characteristic representation of the bird sounds.

Chroma features [49] are commonly used to describe the frequency distributions of different tones in music without considering their specific positions. By viewing bird songs as high-frequency sound signals [50], the frequency distribution characteristics of tones provide an alternative perspective for identifying bird sounds. Initially, the bird sound signal was divided into frames, and a Fourier transform was performed on each frame to obtain its spectrum. The spectrum was then partitioned into multiple bands, and the average amplitude in each band was calculated, resulting in a 12-dimensional vector that represented the frame chromaticity.

Tonnetz features [51] capture the distribution of audio signals in the pitch space. Given the similarities between bird calls and the harmonic structure of music, Tonnetz features are utilized to capture pitch variations and harmonic characteristics in bird sounds. Mapping bird sound signals onto the Tonnetz space allows the tranformation of complex bird sounds into feature vectors that are easily computable and comparable, facilitating identification and classification. Initially, we performed pitch estimation based on the spectral information contained in bird sounds. Then, we mapped the pitch information to the Tonnetz space. Tonnetz features were then computed based on the connections, distances, and angles between the nodes in the Tonnetz space, resulting in a Tonnetz vector.

According to [30], the combination of these three features yields excellent bird sound recognition performance. According to their experimental results, using a log-mel spectrogram alone as an input achieved an accuracy of 88.7%, using MFCCs alone as inputs achieved an accuracy of 88.1%, and using a combination of MFCC-CST features as inputs resulted in an accuracy of 90.1%. The experimental results demonstrated that combining features could provide a more comprehensive representation, leading to higher accuracy in bird sound recognition tasks. However, they also concluded that combining too many features may introduce redundancy and result in a decrease in recognition accuracy. Based on their research, we combined MFCC, Chroma, and Tonnetz features to obtain a multidimensional perspective for bird sounds. However, to address the redundancy in the acoustic feature combination [30], it was necessary to reduce the dimensionality of the resulting high-dimensional features using principal component analysis (PCA) [52] before inputting them into the transformer encoder. This process generated a more compact feature vector, enhancing computational efficiency without sacrificing the representational power of the features.

### 2.4. Deep Feature Extraction and Feature Fusion

**Figure 4:**
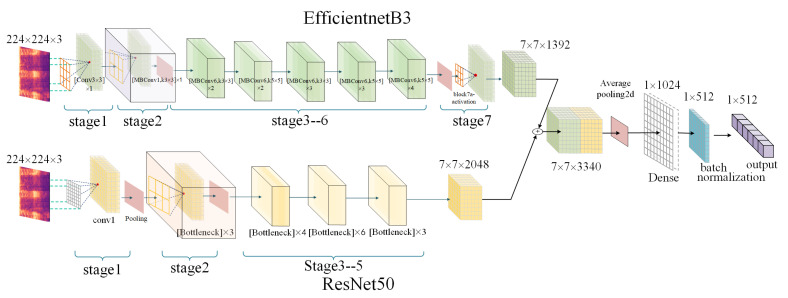
The process of deep feature extraction conducted by using log-mel spectrograms as inputs.

As illustrated in Figure 4, the manually extracted log-mel spectrograms were used as inputs for the pretrained EfficientNetB3 [37] and ResNet50 [38] networks. The outputs of each network were then fused to obtain a set of deep feature vectors. We retained the feature extraction layer of the EfficientNetB3 model while discarding its top network. The features extracted by both CNN-based networks were fused, and an average pooling layer was applied to compute the average of each channel in the matrix, resulting in a feature vector with a size of (7, 7, 3340). To reduce the dimensionality of the fused deep feature vector, a dense layer was added, followed by a normalization layer. The normalization layer served multiple purposes, including accelerating the network training and convergence processes, controlling gradient explosion, and preventing gradient vanishing [53]. Finally, a set of deep feature vectors with a size of (1, 512) was obtained.

The manually extracted MFCC features had a size of (20, 87), the chroma features had a size of (12, 87), and the Tonnetz features had a size of (6, 87). Initially, we flattened and concatenated these features to create a feature vector with a size of (1, 3306). To reduce the dimensionality of the features and obtain a more compact representation, we applied PCA, resulting in a feature vector with a size of (1, 512). We fed the reduced-dimensional feature vector as input into the improved transformer encoder to extract another set of deep features.

### 2.5. The Improved Transformer Encoder

**Figure 5:**
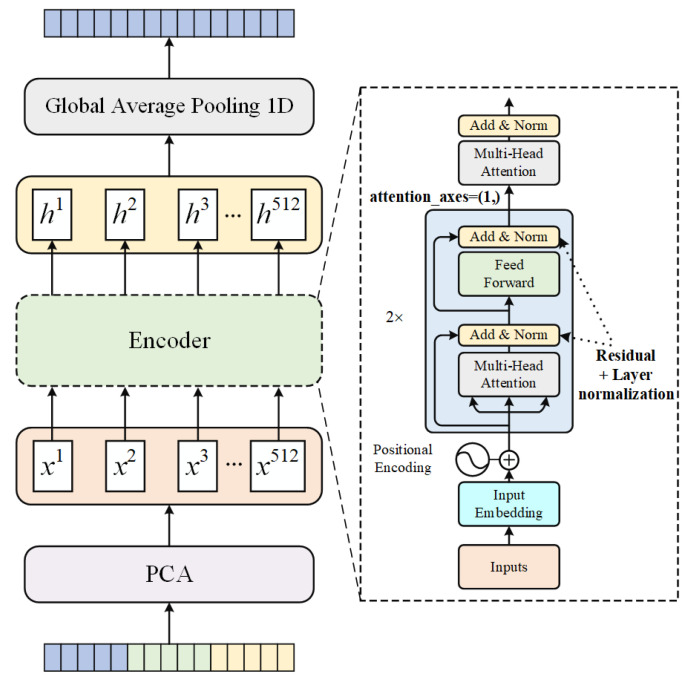
The process of encoding a combination of multiple acoustic features into a set of feature vectors using a transformer encoder.

The transformer encoder consisted of multiple blocks, each containing several multiheaded attention layers and feedforward layers. Before inputting the reduced-dimensional feature vector into the encoder, we included position encodings. This involved utilizing an embedding layer to encode the input as a 512-dimensional vector and then applying a multihead attention layer to perform self-attention calculations on the position encoding layer output. Residual operations and normalization processes were also applied. Next, we employed a feedforward layer to perform nonlinear mapping on the features and applied another round of residual operations and normalization. Given the strong temporal correlations exhibited by bird sound features [7], we paid special attention to the spatial positional relationships between the features during bird sound processing. To capture the global and local relationships of bird sound features more effectively, we introduced a new multiheaded attention layer that specifically focused on the positional axis. The newly added attention layer employed only the positional dimension of attention, enabling the re-encoding of the spatial positional information among the features. This enhanced the acquisition of the spatial positional relationships either between features or within them. The output of the new multiheaded attention layer was updated and served as the input for the subsequent block. After completing the encoding process, we utilized a GlobalAveragePooling1D layer to conduct pooling operations on the final output and finally obtained another set of deep feature vectors of size (1, 512) that contained the positional information between the features. The specific process is illustrated in Figure 5.

## 3. Experiments and Results

### 3.1. Settings

The proposed model combined the log-mel spectrogram feature set with the MFCC, Chroma, and Tonnetz features, as described in Section 2.3. The true category labels were used as the reference output. EfficientNetB3 and ResNet50 were trained to extract the fused features by optimizing the stochastic gradient descent (SGD) process for log-mel spectrogram features [54]. To prevent overfitting, a dropout probability of 0.3 was applied to each fully connected layer. During training, a batch size of eight and an initial learning rate of 0.01 were used. If the validation loss did not improve for two consecutive training rounds, the learning rate was reduced by a factor of 0.7. The transformer encoder was configured with eight multiheaded self-attention layers and two stacked layers. The feedforward layer was utilized for nonlinear feature mapping. In the subsequent classification stage, the LightGBM served as the classifier, as it was designed explicitly for multiclassification tasks. The decision trees in the LightGBM were configured with 31 leaf nodes, and the learning rate was set to 0.01. The number of categories was individually determined for each dataset. To ensure the validity and impartiality of the experiments, a consistent configuration, as summarized in Table 2, was applied across all trials.

### 3.2. Model Evaluation

We evaluated the obtained results using various evaluation metrics, including the accuracy, recall, F1 score, and Precision measures.

Accuracy: This is the ratio of the number of samples correctly classified by the model to the total number of samples. It provided an assessment of the overall correctness of the model’s classification results and was calculated as follows:

(3) $$Accuracy=\frac{TP+TN}{TP+TN+FP+FN}.$$

Recall: This is the ratio of the number of samples correctly predicted by the model as positive cases to the number of true-positive samples. It was used to assess the true-positive case coverage of the model and was calculated as follows:

(4) $$Recall=\frac{TP}{TP+FN}.$$

F1 score: This is the sum of the precision and recall of the model. It was used to assess the comprehensive performance of the model and was calculated as follows:

(5) $$F1=\frac{2*Precision*Recall}{Precision+Recall}.$$

Precision: This is the proportion of samples correctly classified as positive out of all samples labeled as positive:

(6) $$Precision=\frac{TP}{TP+FP}.$$

The accuracy, recall, F1 score and precision range from 0 to 1, with higher values indicating better model performance.

Additionally, we employed the log loss to assess the classification performance of the tested models. A smaller log loss indicates a closer match between the model’s predicted probabilities and the actual labels.

(7) $$logloss=-\frac{1}{N}\sum_{i=1}^{N}\sum_{j=1}^{K}y_{i,j}\log y_{\hat{i},j}.$$

*K* is the number of categories, the actual label of sample *i* is $y_{i,j}$, and the probability predicted by the model is $y_{\hat{i},j}$ ($0\leq y_{\hat{i},j},j\leq 1$).

### 3.3. Results

#### 3.3.1. Model Performance

**Figure 6:**
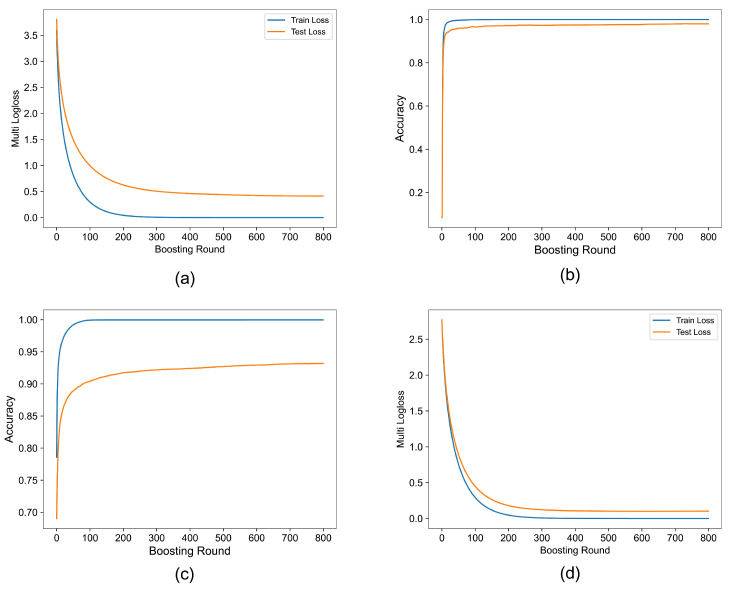
The figure illustrates the accuracy and loss curves obtained during the training and testing phases on two datasets. (**a**) The multilog loss curve of CBC. (**b**) The accuracy curve of CBC. (**c**) The multilog loss curve of Birdsdata. (**d**) The accuracy curve of Birdsdata.

During the training and validation processes, the accuracy curves of the bird sound recognition method proposed in this study exhibited a rapid increase followed by convergence and stabilization, as illustrated in Figure 6. For the Birdsdata dataset, a testing set consisting of 2862 bird song sound fragments was utilized, yielding a stable testing accuracy of 97.99% and a consistent log loss of approximately 0.1032. Similarly, on the CBC dataset, which employed a test set comprising 22,857 bird sound fragments, the accuracy stabilized at 93.18% with a consistent log loss of approximately 0.6816. These experimental results provide strong evidence supporting the superior performance of the proposed method in bird sound recognition. To further evaluate the effectiveness of the model, additional evaluation metrics were employed. On the Birdsdata dataset, the recall rate reached 96.14%, the F1 score reached 96.88% and the precision reached 97.97%. Similarly, on the CBC dataset, the recall rate reached 92.43%, the F1 score reached 93.14% and the precision reached 93.25%, as shown in Table 3. These results further validate the effectiveness of the proposed method in bird sound recognition and demonstrate its robustness in complex acoustic environments.

**Figure 7:**
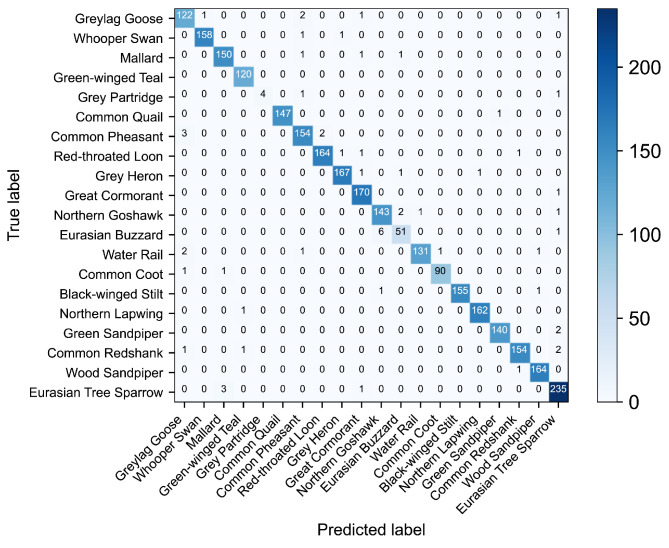
The confusion matrix (Birdsdata).

**Figure 8:**
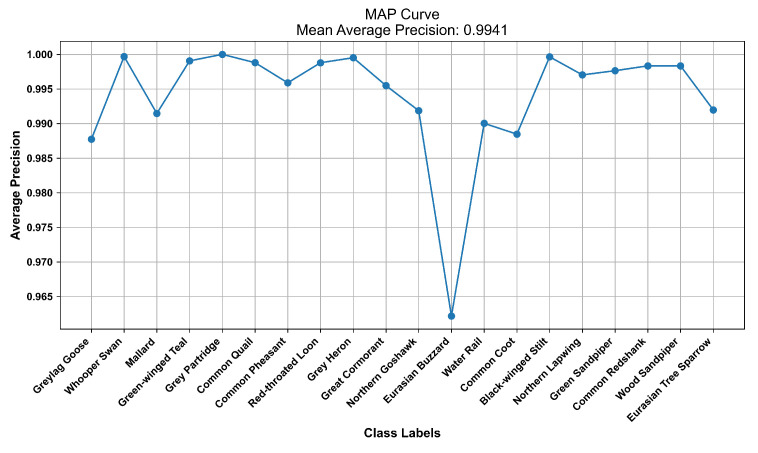
The mean average precision curve (Birdsdata).

**Figure 9:**
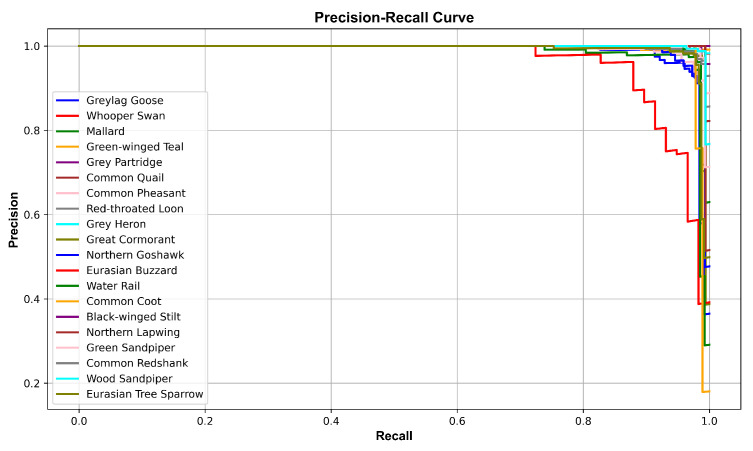
The precision–recall curve (Birdsdata).

During the testing phase, the predicted and actual labels of each sample were recorded. A multiclass confusion matrix was generated using Birdsdata as an example, as presented in Figure 7. This confusion matrix enabled the calculation of accuracy, recall, and F1 score values for each bird species, as outlined in Table 4. Notably, the recall and F1 scores obtained for the gray partridge and Eurasian buzzard were considerably lower than those produced for the other birds. Specifically, the recall rate achieved for the gray partridge was 66.7%, accompanied by an F1 score of 80.0%. In contrast, the Eurasian buzzard demonstrated a recall rate of 87.9% and an F1 score of 90.3%, as depicted in Table 4. This could be attributed to the relatively small numbers of training samples available for these two birds compared to the other species, as well as their less distinctive bird sound features, resulting in lower overall scores. Figure 8 displays the average precision curve obtained on the Birdsdata dataset, featuring an MAP of 0.994. Furthermore, Figure 9 illustrates the precision–recall curve for the same dataset, effectively providing a visual representation of the model’s remarkable bird sound recognition ability.

#### 3.3.2. Comparisons

This study conducted comparative experiments on the different datasets to demonstrate the superior bird sound recognition performance of the proposed method. For the CBC dataset, Gupta et al. [41] selected 100 bird species for identification and classification, achieving an accuracy of 70%. The state-of-the-art (SOTA) method, applied to the CBC dataset [58], utilizes Gammatone frequency cepstral coefficient (GFCC) features as inputs for K-nearest neighbor (KNN) classification. It achieves a peak accuracy of 78.32% in recognizing 264 distinct bird species. In contrast, our proposed method achieved a 93.18% accuracy in validating 264 bird species on the CBC dataset. The AMResNet model [30], identified as one of the SOTA models in the Birdsdata dataset, attained a 92.6% accuracy. Notably, our method outperformed it with a 5.39% improvement in accuracy. Furthermore, Tang et al. [26] attained an accuracy of 94.6% on the Birdsdata dataset by employing a visual transformer with superhead attention (ViT) and MFCCs. In contrast, our method demonstrated a 3.39% improvement in accuracy.

To comprehensively compare our approach with state-of-the-art methods in bird sound recognition, we applied BirdNET and AMResNet, which are considered to have superior bird sound recognition performance [30,57], to the Birdsdata dataset and the CBC dataset. And we used the features detailed in their respective articles as inputs as shown in Table 3, applying the BirdNET model with manually extracted acoustic spectrograms as input features [57]. On the Birdsdata dataset, we achieved an accuracy of 86.7%, a recall of 87.9%, an F1 score of 86.3%, and a precision of 86.5%. Similarly, on the CBC dataset, we obtained an accuracy of 68.3%, a recall of 66.5%, an F1 score of 68.1%, and a precision of 67.9%. The AMResNet model, coupled with manually extracted log-mel spectrogram, Chroma, Spectral contrast, and Tonnetz as input features [30], achieved an accuracy of 88.7%, a recall of 88.0%, an F1 score of 88.5%, and a precision of 88.1% on the Birdsdata dataset. Similarly, on the CBC dataset, it yielded an accuracy of 82.1%, a recall of 82.4%, an F1 score of 82.1%, and a precision of 81.9%. Notably, our proposed method in this paper outperforms BirdNET and AMResNet on both datasets.

Furthermore, we compared our method with the mainstream networks that are commonly used in bird sound recognition. As demonstrated in Experiments 1–5 in Table 3, by fusing the features extracted by EfficientNetB3 and ResNet50, we achieved better results than those obtained using the features extracted by a single network. Experiments 5–6 in Table 3 show that the LightGBM outperformed traditional classifiers such as softmax in terms of classification performance. A comparison among Experiments 7, 8, and 10 in Table 3 revealed that utilizing the transformer encoder as an encoder for the feature set consisting of MFCC, Chroma, and Tonnetz features led to better recognition results. As shown in Experiments 9–11 of Table 3, the amalgamation of these three feature types, as detailed in reference [30], exhibited superior performance to that achieved using only two features. Additionally, these experiments illustrate that improper feature combinations introduce redundancy, leading to a decline in bird sound recognition performance. Overall, the proposed method in this paper achieved the most favorable recognition results.

## 4. Discussion

Birds, being highly active species in ecosystems, serve as crucial biodiversity indicators. Therefore, accurate and swift bird recognition is essential in the context of biodiversity loss [59,60,61]. Bird sound recognition, which provides quantitative information for ecological conservation and management decisions, offers an accurate bird recognition approach. In this study, we proposed a bird sound recognition method that combines two CNN-based networks and a transformer encoder, incorporating log-mel spectrograms and other three acoustic features. Additionally, the method incorporates feature fusion as well as quantile noise estimation [18], aiming to achieve accurate bird sound recognition in real-world environments [14].

The experimental results of our study indicated that using log-mel spectrograms along with MFCC, Chroma, and Tonnetz features could achieve the best performance. This is because log-mel spectrograms, as indicated in [62], have significant potential for addressing the challenge of bird sound recognition by capturing detailed information about the distinctive features that are present in both high- and low-frequency ranges. Previous studies [63,64,65] demonstrated the effectiveness of combining multiple features for accurately recognizing various targets. In this study, we manually extracted log-mel, MFCC, Chroma, and Tonnetz features as model inputs. The log-mel spectrograms of bird sounds were fed into both EfficientNetB3 and ResNet50. The resulting features output by these two networks were then fused to derive a set of deep feature vectors. Simultaneously, the MFCC, Chroma, and Tonnetz features were inputted into the improved transformer encoder, generating an additional set of deep feature vectors. Then, these two sets of deep feature vectors were fused and fed into a LightGBM for recognition. We also considered the redundancy introduced by combining multiple features. To mitigate this, we employed principal component analysis (PCA) to reduce the dimensionality of the high-dimensional data that included these features. This dimensionality reduction method enhanced the computational efficiency and effectiveness of the combined features. By incorporating these deep features, we comprehensively captured the time-frequency domain characteristics of bird sounds, utilizing the complementary information conveyed in different frequency and time dimensions. The findings in [66] indicate that using multiple networks as feature extractors for log-mel spectrograms yields better results than using a single network alone. Our results also showed that the fusion of the features extracted by two CNN-based networks and the usage of an improved transformer encoder lead to better performance. Additionally, the feature fusion steps enhanced the comprehensiveness of the information contained in the features, facilitating the comprehensive analysis of bird sounds and ultimately improving the recognition accuracy of the model.

The results presented in Table 3, from Experiments 1–11, illustrate the significant influence of model choice and feature selection on the performance of bird sound recognition. In addition to comparing with mainstream models, we applied the method described in [30,57] to both the Birdsdata and CBC datasets, with the results presented in Experiments 12 and 13 in Table 4. Experimental results demonstrate that the method proposed in this paper exhibits improvements in accuracy, recall, F1 score, and precision when compared to the methods described in [57] (BirdNET) and [30] (AMResNet). For BirdNET [57], incorporating residual structures enhances the network’s depth. However, the inclusion of downsampling before each residual structure diminished recognition performance when dealing with the short bird sound segments utilized in this study [30]. For AMResNet, the introduction of the residual structure and the attention layer makes it effective in bird sound recognition [30]. However, the channel attention module and the spatial attention module within the attention layer struggle to capture global feature relationships [67], unlike the transformer encoder based on the self-attention mechanism. This limitation hinders the achievement of high recognition accuracy.

Our method combines the strengths of CNN-based networks in terms of spectrum feature extraction [7] with the transformer’s ability to handle long sequences [26,28,68], thus achieving the best performance in comparison with other models. The self-attention mechanism, a key concept in transformers, plays a crucial role in establishing relationships between the positions in the input sequence, enabling global contextual encoding for the feature vectors. By utilizing self-attention, the transformer allows for information interaction and association across the entire input sequence, overcoming the sequential processing limitations and the step-by-step passing operations of traditional sequence models, as demonstrated in [28,68]. Additionally, we enhanced the transformer encoder by introducing a new multiheaded attention layer and specifying the attention mechanism to perform self-attention computations on the spatial positional relationships between features. We believe that the encoding approach of the transformer, compared to that of convolutional neural networks, enables the capture of the spatial positional relationships between discontinuous features in bird sounds. This enhancement further improves the recognition performance of the proposed model.

Accurate bird sound recognition allows us the gaining of insights into the biodiversity and distribution of birds in a specific area, enabling the assessment of biodiversity statuses and changes. Such monitoring aids in identifying endangered species, monitoring the health of ecosystems, and formulating suitable conservation measures. Given the high sensitivity of birds to environmental changes, bird sound recognition allows us the assessment of environmental quality and implementation of timely measures for ecosystem protection. As birds play a crucial role in the ecosystem by controlling insect populations and facilitating seed dispersal, which are vital for maintaining ecological balance, identifying bird sounds enables us to gain a deeper understanding of their ecological functions.

Although the proposed bird sound recognition method shows promising performance and profound implications, challenges still need to be addressed. Bird sounds exhibit various frequency distributions and display continuous time-varying characteristics [7]. The high variability of bird sounds within the same species across different areas adds complexity to the bird sound recognition task. Additionally, the limited availability of large-scale standardized datasets for training and evaluation in bird sound recognition tasks poses challenges [69]. To address these issues, it is crucial to further enhance the model’s generalization ability by collecting a more extensive range of bird sound data from diverse regions before the method can be extended and practically applied to bird population detection and biodiversity conservation.

## 5. Conclusions

This study was the first to propose a bird sound recognition method based on multiple CNN-based networks and a transformer encoder. It focused on addressing the key aspects of bird sound recognition, including noise estimation and denoising, silence detection, feature extraction, fusion, and classification.

The proposed method incorporated quantile noise estimation to estimate environmental noise and eliminate interference in bird sound recognition tasks. After performing silence detection and segmenting audio fragments, we employed EfficientNetB3 and ResNet50 to extract the deep features of manually extracted log-mel spectrograms. Additionally, we integrated EfficientNetB3 and ResNet50 and the improved transformer encoder to maximize their strengths and capture the distinctive characteristics of bird sounds. The proposed method demonstrated outstanding performance on the two publicly available datasets that we used. Specifically, on the Birdsdata dataset, we achieved an accuracy of 97.99%, a recall of 96.14%, an F1 score of 96.88% and a precision of 97.97%. On the CBC dataset, we achieved an accuracy of 93.18%, a recall of 92.43%, an F1 score of 93.14% and a precision of 93.25%. These results demonstrated the superiority of our proposed method over other methods. The improved bird sound recognition accuracy of this study has profound implications. Bird sound recognition holds significant importance in ecology, as it aids in monitoring biodiversity, assessing environmental quality, and maintaining ecological balance. Overall, the method presented in this paper significantly contributes to current biodiversity conservation efforts by providing a swift and accurate method for recognizing birds through their sounds.

## Figures and Tables

**Table:** Detailed partitioning strategy used for the training, validation and testing sets of two datasets.

Dataset	Training Set (Classes)	Validation Set (Classes)	Testing Set (Classes)
Birdsdata	8587 (20)	2862 (20)	2862 (20)
CBC	68,573 (264)	22,857 (264)	22,857 (264)

**Table 2:** Settings.

Designation	Parameters
CPU	13th Gen Intel Core i7-13700KF
Memory	32 GB DDR5
GPU	NVIDIA GeForce RTX 3070
System platform	Windows 10
Software environment	Tensorflow-gpu 2.8.0, Keras 2.8.0
	Cuda 11.4, Anaconda 3

**Table 3:** Comparison with the results of other models on the Birdsdata and CBC datasets.

Experiment	Model	Features	Accuracy			Recall			F1 Score			Precision	
			**Birdsdata**	**CBC**		**Birdsdata**	**CBC**		**Birdsdata**	**CBC**		**Birdsdata**	**CBC**
1	ResNet50+softmax [38]	log-mel	94.5%	61.2%		94.1%	60.3%		91.0%	60.5%		94.4%	62.0%
2	EfficientNetB3+softmax [37]	log-mel	95.2%	76.5%		91.5%	76.1%		92.6%	74.3%		92.8%	74.5%
3	DenseNet121+softmax [55]	log-mel	93.5%	70.9%		93.1%	70.2%		90.7%	68.8%		91.7%	71.3%
4	VGG16+softmax [56]	log-mel	94.2%	64.1%		92.1%	61.5%		90.8%	63.9%		92.8%	64.0%
5	EffcientNetB3+ResNet50+softmax	log-mel	96.0%	81.3%		93.6%	80.7%		94.1%	80.2%		94.8%	81.3%
6	EffcientNetB3+ResNet50+LightGBM	log-mel	97.1%	90.8%		96.0%	89.2%		93.9%	90.6%		93.8%	90.2%
7	EffcientNetB3+ResNet50+softmax	MFCC+Chroma+Tonnetz	85.6%	71.4%		85.5%	66.3%		83.9%	68.7%		85.0%	67.3%
8	LightGBM [39]	MFCC+Chroma+Tonnetz	88.5%	81.2%		86.3%	81.2%		87.6%	79.8%		88.6%	81.5%
9	Transformer encoder+LightGBM [28,39]	Chroma+Tonnetz	81.6%	78.5%		82.3%	76.9%		82.9%	76.6%		82.9%	77.0%
10	Transformer encoder+LightGBM	MFCC+Chroma+Tonnetz	89.4%	83.1%		89.2%	82.9%		88.5%	80.2%		89.6%	80.5%
11	Transformer encoder+LightGBM	MFCC+Chroma+Tonnetz+Spectral contrast	88.5%	83.5%		89.0%	81.7%		87.9%	82.2%		89.3%	82.1%
12	BirdNET [57]	spectrogram	86.7%	68.3%		87.9%	66.5%		86.3%	68.1%		86.5%	67.9%
13	AMResNet [30]	log-mel+Spectral contrast+Chroma+Tonnetz	88.7%	82.1%		88.0%	82.4%		88.5%	82.1%		88.1%	81.9%
14	Methodology of this article	log-mel+MFCC +Chroma+Tonnetz	**98.0%**	**93.2%**		**96.1%**	**92.4%**		**96.9%**	**93.1%**		**97.8%**	**93.3%**

**Table 4:** The model results obtained for each bird species in the Birdsdata dataset.

Classes	Accuracy (%)	Recall (%)	Precision (%)	F1 Score (%)	Samples
Gray Goose	99.6	96.1	94.6	95.3	127
Whooper Swan	99.8	98.7	99.4	99.0	160
Mallard	99.7	98.0	97.4	97.7	153
Green–Winged Teal	99.9	100	98.4	99.1	120
Grey Partridge	99.9	66.7	100	80.0	6
Common Quail	99.9	99.3	100	99.6	148
Common Pheasant	99.6	96.8	96.2	96.5	159
Red-Throated Loon	99.8	98.2	98.8	98.5	167
Gray Heron	99.8	98.2	98.8	98.5	170
Great Cormorant	99.8	99.4	97.1	98.3	171
Northern Goshawk	99.6	97.3	95.3	96.3	147
Eurasian Buzzard	99.6	87.9	92.7	90.3	58
Water Rail	99.6	96.3	98.2	95.3	136
Common Coot	99.9	97.8	98.9	98.3	92
Black–Winged Stilt	99.9	98.7	100	99.3	157
Northern Lapwing	99.9	99.4	99.3	99.4	163
Green Sandpiper	99.9	98.6	99.3	98.9	142
Common Redshank	99.8	97.5	98.7	98.0	158
Wood Sandpiper	99.9	99.4	98.8	99.0	165
Eurasian Tree Sparrow	99.5	98.3	96.3	97.3	239

## Data Availability

Not applicable.
